# Intercomparison of crop establishment methods for improving yield and profitability in the rice-wheat system of Eastern India

**DOI:** 10.1016/j.fcr.2020.107776

**Published:** 2020-05-01

**Authors:** Madhulika Singh, Pankaj Kumar, Virender Kumar, I.S. Solanki, Andrew J. McDonald, Ajay Kumar, S.P. Poonia, Vipin Kumar, Anurag Ajay, Anurag Kumar, Deepak K. Singh, Balwinder-Singh, Sudhanshu Singh, Ram K. Malik

**Affiliations:** a International Maize and Wheat Improvement Centre, NASC Complex, New Delhi, India; b International Rice Research Institute, Los Baños, Philippines; c Indian Council of Agriculture Research, Pusa, New Delhi, India; d International Rice Research Institute, NASC Complex, New Delhi, India; e Soil and Crop Sciences Section, School of Integrative Plant Sciences, Cornell University, Ithaca, NY, USA

**Keywords:** DSR, Directly sown rice, MTR, Machine transplanted rice in non-puddled soil, PTR, Puddled transplanted rice, SRI, System of rice intensification, ZT, Zero-tillage, CT, Conventional tillage, Machine transplanting, Non-puddled rice, Puddled transplanted rice, System of rice intensification, System of wheat intensification, Zero-tillage

## Abstract

Conventionally managed rice-wheat systems of the eastern Indo-Gangetic Plains (E-IGP) that rely on soil puddling for rice and intensive tillage for wheat are low-yielding and resource-inefficient, leading to low profitability. While a host of alternative tillage and crop establishment (TCE) methods have been advocated as solutions for sustainably enhancing productivity and profitability, few systematic comparisons of these methods are reported. To address this gap, a three-year field study was conducted in Bihar, India with the goal of identifying TCE methods for rice-wheat systems that are high yielding, less resource-intensive, and more profitable. The following systems were evaluated: 1) puddled transplanted rice (PTR) followed by (fb) conventional tillage wheat (CTW) or zero-tillage wheat (ZTW); 2) machine transplanted rice in non-puddled soil (MTR) fb ZTW; 3) the system of rice intensification (SRI) fb system of wheat intensification (SWI); and 4) dry-seeded rice (DSR) fb ZTW. Rice cultivar duration (short versus medium-duration) was incorporated as a subplot treatment in all systems. Rice yields were similar with all methods, except DSR yield was 11 % lower and MTR yield was 7% higher than PTR in the third year. Cost of production was US$ 149 and 77 ha^−1^ lower in DSR and MTR, respectively, and US$ 84 ha^-1^ higher in SRI than PTR. The gross margin and benefit-cost (B:C) ratio was highest in MTR followed by DSR and lowest in SRI. In wheat, ZT resulted in a higher yield than CTW, especially when ZTW was cultivated after non-puddled rice (e.g., DSR or MTR). ZTW reduced production costs by US$ 69 ha^-1^, whereas SWI increased it by US$ 139 ha^-1^ relative to CTW. The higher yield and lower cost of production resulted in a higher gross margin (US$ 82−355 ha^−1^ and US$ 129−409 ha^−1^ higher than CTW and SWI, respectively) and a higher B:C ratio in ZTW treatments than CTW and SWI. At the system level, MTR or DSR followed by ZTW had both superior crop yields and consistently higher gross margins (US $133 to 382 ha^-1^) than other practices. On the other hand, the SRI fb SWI system had no yield advantage and poorer economic performance than conventional practices. In all systems, the inclusion of a medium-duration rice hybrid resulted in higher rice and system yields. These results suggest that significant gains in profitability are possible with emerging TCE practices in rice-wheat systems, but alternatives such as the SRI and SWI will likely erode farmer incomes.

## Highlights

• DSR or MTR followed by ZT wheat gave higher system yield and gross margin.
• Production cost reduced by US$ 149 and 77 ha^−1^ in DSR and MTR, respectively and increased by US$ 84 ha^−1^ in SRI than in PTR.
• In wheat, ZT had higher yield and lower production cost (US$ 69 ha^−1^) but SRI increased cost by US$ 139 ha^−1^ than CT.

## 1 Introduction

The rice-wheat cropping system of the Indo-Gangetic Plains (IGP) is practiced on approximately 10.3 million ha in India (Timsina and Connor, 2001). The Indian western IGP (WIGP; Punjab, Haryana, western Uttar Pradesh) has seen broad-scale adoption of ‘Green Revolution’ advances in crop genetics and agronomic management; high yields of rice and wheat are common, and crops are fully irrigated with a high level of mechanization and input use, but evidence of natural resource degradation and depletion is accelerating (Aggarwal et al., 2004; Erenstein et al., 2007). In contrast, the Indian eastern IGP (EIGP; Eastern Uttar Pradesh, Bihar, and West Bengal) is better endowed with natural resources (e.g., abundant groundwater) but has not achieved similar levels of input use and productivity. The region is characterized by a high density of rural poverty and food insecurity with large yield gaps for cereal staples (rice and wheat), landholdings are small and fragmented, irrigation infrastructure is less developed, climatic aberrations (e.g., flood and drought) are more frequent, and extension and market institutions are weaker (Laik et al., 2014). As a result, there is increased interest of the Indian Government to increase staple food production and productivity in the EIGP as a development imperative through flagship programs such as ‘Bringing Green Revolution to Eastern India’ (BGREI, http://bgrei-rkvy.nic.in), the National Food Security Mission (NFSM), and Rashtriya Krishi Vikas Yojna (RKVY).

Prevailing crop establishment methods in the EIGP for rice and wheat are resource inefficient and costly. Rice is commonly grown under rainfed or limited-irrigation conditions during the monsoon season by transplanting rice seedlings into puddled (wet-tillage) soil (PTR).. In the EIGP, seedling nursery establishment is typically initiated with the onset of the monsoon. Moreover, rainfall breaks and labor availability bottlenecks often result in asynchrony between the optimal seedling age and the ability of farmers to puddle and transplant their fields. Both factors often delay rice transplanting beyond the optimal planting window, resulting in yield loss of rice and of the succeeding wheat crop if rice harvest prevents timely wheat sowing (Balwinder-Singh et al., 2019). Recently, PTR has also become less profitable because it is highly resource-intensive (e.g., labor, water, and energy – all of which are becoming scarce and expensive) (Kumar and Ladha, 2011; Kumar et al., 2018). Similarly, conventional method of wheat establishment is resource-intensive and typically consists of multiple tillage passes to create a friable seedbed. This often leads to a long turnaround period. The late harvest of rice (due to late planting of rice) combined with the long turnaround period associated with conventional tillage often results in the late planting of wheat, leading to low wheat productivity. Late planting has been a major constraint in improving the wheat productivity in EIGP with a yield reduction of 27.6 kg per day per hectare if planting is delayed beyond mid November (normal time) (Tripathi et al., 2005).

Several alternative crop establishment methods have been evaluated in the IGP such as mechanized dry-seeded rice (DSR) (Jat et al., 2014; Kumar et al., 2018; Laik et al., 2014), mechanical transplanted rice in non-puddled soil (MTR) (CSISA, 2015), the system of rice intensification (SRI) (Thakur et al., 2010;), zero-tillage wheat (ZT) (Keil et al., 2015), and the system of wheat intensification (SWI) (Raj et al., 2017). Many studies demonstrate that DSR is more labor and water-efficient and more profitable than PTR (Gathala et al., 2013; Kumar and Ladha, 2011; Kumar et al., 2018; Laik et al., 2014; Sudhir-Yadav et al., 2011a, 2011b). In terms of yield, the results are variable, with some reports of similar yields (Gathala et al., 2013; Kumar et al., 2015; Laik et al., 2014; Sudhir-Yadav et al., 2011a,b), while others reported lower yields for DSR (Jat et al., 2009; Kumar et al., 2018; Kumar and Ladha, 2011; Ladha et al., 2009; Saharawat et al., 2010). Similarly, some studies have found MTR (CSISA, 2015) and SRI (Fernandes and Uhoff, 2002; Raj et al., 2017) to be more productive and profitable than PTR. However, for SRI, many studies reported no yield gain from SRI compared to conventional best agronomic practices (Doberman, 2004; McDonald et al., 2006; Sheehy et al., 2005; Suryavanshi et al., 2012).

At the cropping systems level, both DSR and MTR not only address the issues of labor scarcity and the rising cost of cultivation by avoiding puddling and reducing labor requirements but also bring opportunity for early rice establishment, as less water is needed for these methods and can be achieved by utilizing pre-monsoon rainfall or supplemental irrigation (Kar et al., 2018). This also allows the timely establishment of a succeeding wheat crop, leading to higher system productivity. In wheat, ZT has been adopted at scale in WIGP and is now gaining momentum in EIGP because of the clear positive impact it offers on productivity, profitability, environmental sustainability, and resilience to heat stress (CSISA, 2015; Erenstein and Laxmi, 2008; Keil et al., 2015). Recently, a few development organizations have started evaluating SWI in India. Very limited results are published on the performance of SWI relative to CT and ZT wheat.

Few studies have conducted a side-by-side comparison of all four rice establishment methods at the same time for yield, profitability, and impact at the cropping system level, which has limited the ability to make robust recommendations about the relative merits of each approach. The present study was conducted with the objective of comparing the relative performance of rice and wheat establishment methods on yields, cost of cultivation, and profitability at an individual crop (rice and wheat) and cropping system (rice + wheat) level. We hypothesized that mechanized non-puddled rice (DSR and MTR) followed by ZT wheat will results in similar or higher system yields with a lower cost of cultivation which will result in high net income compared to manual puddled transplanted methods (PTR and SRI) followed by conventional tillage wheat.

## 2 Material and methods

### 2.1 Experimental site

**Fig. 1:**
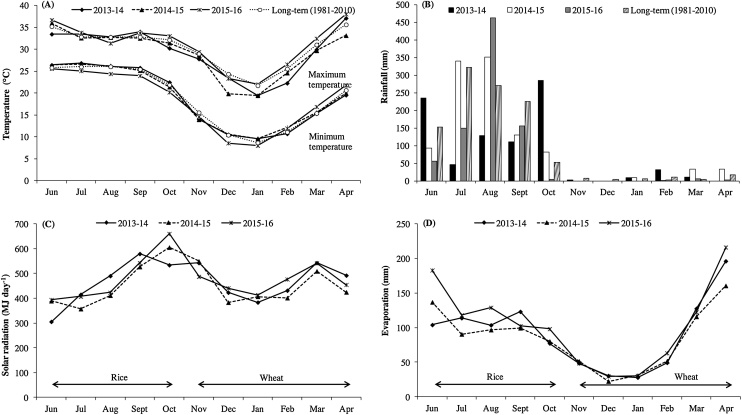
Monthly average daily maximum and minimum temperature (A), monthly rainfall (B), monthly mean daily solar radiation (C), and monthly average evaporation (D) during the study years 2013-14 to 2015-16 and long term average (1981-2010). The rice phase was from June to October, and the wheat phase was from November to mid-April.

A field experiment was carried out with rice-wheat rotation for three years from 2013-14 to 2015-16 at the Regional Research Station of Indian Agricultural Research Institute, Pusa, Samastipur, Bihar, India 25°99′N and 85°66′E. The climate of the area is subtropical humid with an average annual rainfall of 1250 mm (75–80 % of which is received during June-September), an average relative humidity of 50–70 % across the year, a daily minimum temperature of 5−8 °C in January, and a maximum daily temperature of 40−45 °C in June. The weather during the study years is presented in Fig. 1. Based on the initial soil analysis done at the beginning of the experiment (0−15 cm layer), the soil at the experimental site has a sandy loam texture with pH 8.4, electrical conductivity (EC) of 0.30 dS m^−1^, organic carbon content of 0.36 % (Walkley and Black), available nitrogen of 116 kg ha^-1^ (Kjeldahl digestion), available P_2_O_5_ of 71 kg ha^−1^ (Olson P; 0.5 M NaHCO_3_ extraction) and available K_2_O of 256 kg ha^−1^ (by emission spectrophotometry; 1 M neutral NH_4_OAC-extractable K).

### 2.2 Treatment details, experimental design, and management

The experiment was laid out in a split-plot randomized complete block design with three replications. The four rice crop establishment methods [puddled transplanted rice (PTR), machine transplanted rice in non-puddled soil (MTR), the system of rice intensification (SRI), and dry-seeded rice (DSR)] were tested in the main plot. Rice cultivars [PRH-10 (shorter duration hybrid – 115 days duration) and Arize-6444 (medium-duration hybrid – 135 days duration)] were applied as subplot treatments. During the wheat season, zero-till (ZT) wheat was planted in the DSR and MTR plots, and system of wheat intensification (SWI) was planted in SRI plots. The PTR plots were divided into two equal parts, and one part was planted with conventional till (CT) wheat and the other with ZT wheat. In the third year, due to non-availability of seeds of the PRH-10 hybrid, this cultivar was replaced with Arize-6129, another short duration hybrid with 115 days duration. The sizes of the main plot and subplots were 23 m  ×  16.5 m and 23 m x 8 m, respectively. Prior to the start of the experiment, the entire experimental area was precisely leveled using a laser land leveler.

Crop management practices deployed for different rice establishment methods are summarized in Table 1A and briefly elaborated here. DSR was seeded using zero-till seed and fertilizer drill. On the same day of DSR sowing, nurseries for PTR, MTR, and SRI were established. Standard practices for nursery raising as recommended for respective crop establishment methods were used. In a mat-type nursery used for MTR, 22 kg ha^−1^ seed were sown on a thin layer (1.3–2.0 cm) of soil (a mixture of sieved soil and farmyard manure in a ratio of 4:1) placed on a perforated polyethene sheet in a raised bed system. The polythene sheet prevents the roots of the rice seedlings from penetrating into the underlying soil and hence creates a dense mat of roots, which can be easily uprooted without damaging roots for mechanical transplanting. For the non-mat nurseries used for PTR and SRI, seed rates of 12 and 5 kg ha^−1^ were sown, respectively. To raise the nursery for SRI, the method described by the Department of Agriculture, Government of Bihar was followed, in which seeds were treated and sown in nursery beds along with the application of 0.125 tonnes of vermicompost to nursery area sufficient for transplanting an area of 1 ha. In SRI, 12-day-old seedlings were transplanted manually, whereas for MTR, 15-day-old seedlings were transplanted in non-puddled soil using an 8-row self-riding type transplanter (VST Pvt Ltd). For PTR, 21-day-old seedlings were manually transplanted in lines. For MTR, the land was prepared with dry tillage (tyne cultivator + planking), and then plots were flooded overnight prior to transplanting to soften the soil, but puddling was not performed; 1−2 cm of standing water was maintained while transplanting. For PTR and SRI, tyne-cultivation and planking were performed when the soil was dry, followed by flooding and wet-tillage using a puddler. For DSR, plots were tyne-cultivated when dry, and then rice was sown. For SRI, we followed all the practices recommended by the Bihar Department of Agriculture, including younger seedlings (12-d old), wider spacing (25 cm x 25 cm), a single seedling per hill, alternate wetting and drying method of water management, adding organic manure such as compost (suggested rate for vermicompost is 1.0 t ha^-1^ if possible), and weed control by a cono-weeder that also facilitates aeration (DOA Bihar, 2019).

**Table 1A:** Summary of crop management practices for different rice establishment methods.

Practice	Puddled transplanted rice (PTR)	Machine transplanted rice (MTR)	System of rice intensification (SRI)	Dry seeded rice (DSR)
Land preparation	Puddling (2 dry-cultivation + wet-tillage (puddling) + leveling)	Nonpuddled (2 dry-cultivation + leveling).	Puddling (same as PTR)	Nonpuddled (2 dry-cultivation + leveling).
Nursery type	Normal	Mat-type	Normal	NA
Seed rate (kg ha^−1^)	12.0	22.5	5.0	20.0
Spacing (cm)*	20.0 × 15.0	23.8 × 17.0	25.0 × 25.0	20.0
Seedling age (days)	21	15	12	NA (directly sown)
Seedlings hill^−1^ (#)	2-3	3-4	1	NA
Establishment method	Transplant manually	Transplant mechanically	Transplant manually	Drill-seeded with seed drill
Weed management	Pretilachlor @ 0.75 kg ai ha^−1^ at 1-3 DAT followed by bispyribac-sodium @ 0.02 kg ai ha^-1^ at 20-25 DAT + 1 spot hand weeding	same as in PTR	Cono-weeding at 15–20 DAT and 30–35 DAT	Pendimethalin @ 1 kg ai ha^−1^ at 1-3 DAS followed by bispyribac-sodium @ 0.025 kg ai ha^−1^ at 20-25 DAS + 1 spot hand weeding
Nutrient Management	N: P_2_O_5_: K_2_O: ZnSO_4_ @ 150:60:40:25 kg ha^−1^	same as in PTR	Same as in PTR +0.275 t ha^−1^ vermicompost in the main field and 0.125 tons in nursery.	same as in PTR
Water management	Irrigation applied with disappearance of water and appearance of hairline crack	Same as PTR	Same as PTR	Irrigation applied with appearance of hairline crack

In all rice plots, full P_2_O_5_, K_2_O, Zn and 1/3rd N were applied at basal levels at the time of planting/sowing in the form of diammonium phosphate (DAP), muriate of potash (MOP), and ZnSO_4_, respectively. For transplanted rice (MTR, SRI and PTR), basal fertilizer (DAP, MOP and Zinc) was applied at final puddling just prior to transplanting. For DSR, these were drilled at the time of planting using a zero-till seed and fertilizer drill. The remaining N was applied in the form of urea in two equal splits at the active tillering and panicle initiation stages. In SRI plots, 0.275 t vermicompost ha^−1^ was also added at the time of puddling. For weed management, pre-emergence herbicide (pendimethalin at 1.0 kg ai/ha for DSR and pretilachlor at 0.75 kg ai/ha for MTR and PTR) was applied 1–3 days after sowing (DAS) or transplanting (DAT) followed by a post-emergence herbicide (bispyribac-sodium at 20 g ai ha^−1^ for PTR and MTR and 25 g ai ha^−1^ for DSR at 20–25 DAS/T). One-hand weeding was also performed to remove any escaped weeds. In SRI, weeds were controlled by using a cono-weeder two times at 15–20 DAT and 30–35 DAT. Disease and insect-pests were managed as per the standard recommended practices on an as-needed basis.

For water management, alternate wetting and drying (AWD) was followed in all treatments. In transplanted treatments (SRI, MTR and PTR), plots were kept flooded (5-cm flood depth) for the first 7–10 days to facilitate crop establishment, and subsequently, AWD was followed with the criterion of irrigation application at the disappearance of floodwater and appearance of a hairline crack (Gathala et al., 2013). For DSR, plots were maintained around field capacity with irrigation applied by the the appearance of a hairline soil cracks. During each irrigation, a flood depth of ∼5-cm was applied.

For the wheat crop, the agronomic practices deployed for different wheat establishment methods are summarized in Table 1B and briefly explained here. Prior to sowing, CT and SWI plots were prepared using conventional tillage practices (tyne cultivator + rotavator), whereas ZT plots were sown without tillage (Table 1B). CT and ZT plots were sown using a zero-till seed and fertilizer drill whereas SWI was sown manually. In ZT plots, prior to sowing, emerged weeds were killed by spraying glyphosate at 1.0 kg ai ha^−1^. Wheat was sown on 1, 6, and 11 November in 2013, 2014, and 2015, respectively. All plots were fertilized at the same rates. Full P and K in the form of DAP and MOP and 23 kg of N (derived from DAP) were applied at sowing using either zero-till seed and fertilizer drill in ZT and CT plots or mixed in the soil with the last tillage in SWI. The remaining N was applied in the form of urea in two equal splits at the first (crown root initiation, CRI) and second (tillering) irrigations. In SWI, vermicompost at 0.275 t ha^−1^ was also incorporated during the last tillage operation. A total of four irrigations were applied in all plots coinciding with critical growth stages (CRI, tillering, flowering, and milk/grain filling stage).

**Table 1B:** Summary of crop management practices for different wheat establishment methods.

Practice	Conventional-till (CT) wheat	Zero-till (ZT) wheat	System of wheat intensification (SWI)
Land preparation	One plowing (2 passes of cultivator) + one rotavator (2 passes)	Glyphosate @ 1.0 kg ai/ha was used as pre-plant 2 days before sowing to kill existing weeds	One plowing (2 passes of cultivator) + one rotavator (2 passes)
Seed rate (kg ha^−1^)	100	100	25
Seed treatment	Not treated	Not treated	Seeds treated with cow urine, jaggery and warm water
Spacing (cm)*	20	20	20 × 20
Establishment method	Mechanically with seed drill	Mechanically with seed drill	Manually by dibbling method
Fertilizer management	N: P_2_O_5_: K_2_O @ 150:60:40 kg ha^−1^	same as CT	same as CT + vermicompost
Weed management	Pre-mix herbicide sulfosulfuron + metsulfuron @ 32 g ai ha^−1^ at 30-35 DAS	same as CT	Cono-weeding was done at 25 and 40 DAS
Water management	4 irrigation was applied coinciding with important crop growth stage and soil moisture status	same as CT	same as CT

Both rice and wheat were harvested manually at a height of 5-7-cm from the ground. All loose residues were removed and anchored residues (∼5-cm) were incorporated during land preparation before rice establishment, whereas during the wheat phase, anchored residues were either incorporated (conventional tillage and SWI) or kept on surface in ZT treatments.

### 2.3 Crop harvest and yield estimation

For rice and wheat yield estimation, an area of 6 m by 21 m (126 m^2^) was manually harvested and mechanically threshed from each plot. For the PTR plots, which were split into two equal plots during wheat, an area of 3 m by 21 m (63 m^2^) was harvested for wheat yield estimation. Grain moisture content was recorded at the time of yield estimation, and final yields of rice and wheat were adjusted to 14 % and 12 % grain moisture content, respectively. For system yield estimation, yields of both rice and wheat were combined.

### 2.4 Economic analysis

For economic analysis, the total variable cost (TC), gross margin (GM), gross return (GR) and cost: benefit (B:C) ratio of each treatment were calculated. For TC, the cost of all inputs was included, such as land preparation, sowing, seed, irrigation water, nutrients, pesticides, harvesting, threshing, and all labor and machinery operations (Table 2). The labor cost was estimated by multiplying labor used in all operations (person-days ha^−1^) with the minimum wage rate as per India’s labor law (Minimum Wage Act, 1948). For machinery costs, rental charges for different operations including land preparation, seeding/transplanting and threshing were used in the economic analysis. Since the exact amount of water was not measured, fixed charges for irrigation water were used irrespective of treatment (INR 12,500 and 15,000 ha^−1^ for short and medium-duration rice varieties, respectively, and INR 3500 ha^−1^ for conventional tillage wheat and INR 4500 ha^-1^ for ZT wheat) as water application criteria were the same across all treatments. However, the extra cost of irrigation was computed for transplant treatments in rice (INR 1500 ha^-1^ for PTR and SRI where the field was puddled and transplanted, and INR 1200 ha^-1^ for MTR where the field was not puddled but flooded overnight to facilitate transplanting). The lower cost of irrigation (INR 1000 ha^−1^) was used for ZT wheat because it is reported by various studies that ZT provides irrigation water saving (20–36 %) compared to conventional tillage wheat, mainly through savings from first irrigation but to some extent in the subsequent irrigations as well, as irrigation water advances quickly in untilled soil than in tilled soil (Hobbs et al., 1997; Mehla et al., 2000; Gupta and Seth, 2007). The GM and B:C ratio were calculated per the equation given below:

GM = GR – TC

where GR was estimated as given below:

GR = Grain yield (rice or wheat) x minimum support price (MSP) of the commodity (rice or wheat) offered by the Government of India in the specific year.

B: C ratio = GR/TC

**Table 2:** Minimum support price of rice and wheat and rates used for calculating costs of key inputs in economic analysis.

Particular	Input cost
Minimum support price for rice (INR kg^−1^)	
*Arize-6444 or 6129*	13.1 (2013); 13.6 (2014); 14.1 (2015)
*PRH-10*	15.1 (2013); 15.6 (2014); 16.1 (2015)
Minimum support price for wheat (INR kg^−1^)	14.00 (2014); 14.50 (2015); 15.25 (2016)
Labor wage (INR person^−1^ day^−1^)	193 (2013-14); 198 (2014-15); 204 (2015-16)
Seed (INR kg^−1^)	
*Wheat*	40
*Rice hybrid (Arize 6444/6129)*	250
*Rice hybrid (PRH-10)*	120
Plowing (2 passes) rental charges (INR)	2750
Puddling rental charges (INR ha^−1^)	4400
Crop establishment rental charges (INR ha^−1^)	
*DSR sowing*	2000
*Mechanical transplanting*	2500
*ZT wheat sowing*	2000
Urea (INR kg^−1^)	6
DAP (INR kg^−1^)	24
MOP (INR kg^−1^)	16
Zinc sulfate (INR kg^−1^)	50
Vermicompost (INR t^−1^)	5000
Irrigation – Diesel pump rental charges (INR hr^−1^)	100

System level GM and TC were calculated by adding the values of both the rice and wheat crops.

### 2.5 Data analysis

The data were subjected to ANOVA and were analyzed using general linear model procedures in Statistical Analysis System (SAS). Data were analyzed using a split-plot design with crop establishment methods as the main plot and rice hybrids as subplots. Treatment means were separated using Tukey’s HSD test (SAS Institute, 2001). Linear contrasts were used to compare single or multiple treatments with other treatments.

## 3 Results

### 3.1 Crop and cropping system yields

#### 3.1.1 Rice

Based on three-year results, rice yields were not affected by the TCE method except in the third year (Table 3). In the first two years, all rice establishment methods had similar yields, whereas in the third year, DSR yield was 11–17% lower than that of other TCE methods. In all three years, the medium-duration hybrid (Arize-6444) gave a higher yield than the shorter-duration hybrid (PRH-10 or Arize-6444). In the first two years, Arize-6444 gave 1.3 to 1.4 t ha^−1^ higher yields than PRH-10, but in year 3, the difference between Arize-6444 and Arize-6129 declined to 0.4 t ha^-1^.

**Table 3:** Rice yields of two cultivars under different rice establishment (CE) methods during 2013-2015 at PUSA in Samastipur, Bihar^1^.

^1^Within column for each year, means followed by the same letter are not different at the 0.05 level of probability using Tukey's HSD test.

*PTR fb CTW = Puddled transplanted rice followed by conventional tillage wheat; MTR fb ZTW = machine transplanted rice followed by zero-tillage wheat; DSR fb ZTW = dry seeded rice followed by zero-tillage wheat; SRI fb SWI = system of rice intensification followed by system of wheat intensification.

** Rice cultivar PRH-10 was used in 2013 and 2014, whereas in 2015, Arize-6129 was used as a short duration hybrid instead of PRH-10.

#### 3.1.2 Wheat

TCE methods as well as the choice of rice cultivar affected wheat yield (Table 4, ANOVA). Puddling during the rice phase significantly influenced the yield of the succeeding wheat crop, and the negative impact of puddling on wheat yield was visible after one cropping cycle (Table 4, see the p-value of contrast). Wheat yield, when grown after puddled rice (PTR and SRI), was 7% and 19 % lower than that obtained when grown after non-puddled rice (DSR and MTR) in years 2 and 3, respectively.

**Table 4:** Wheat yields under different tillage and crop establishment (CE) methods from 2013-14 to 2015-16 at PUSA in Samastipur, Bihar^1^.

^1^Within column for each year, means followed by the same letter are not different at the 0.05 level of probability using Tukey's HSD test.

*PTR fb CTW = Puddled transplanted rice followed by conventional tillage wheat; MTR fb ZTW = machine transplanted rice followed by zero-tillage wheat; DSR fb ZTW = dry seeded rice followed by zero-tillage wheat; SRI fb SWI = system of rice intensification followed by system of wheat intensification.

** Rice cultivar PRH-10 was used in 2013 and 2014, whereas in 2015, Arize-6129 was used as a short duration hybrid instead of PRH-10.

In year 1, all TCE combinations performed similarly except CT wheat after PTR, which gave 12–18 % lower yield than the rest of the treatments (Table 4). In other years, ZT wheat after non-puddled rice (DSR or MTR) gave 12–31 % higher yield than CT wheat or SWI grown after puddled rice (PTR fb CTW or SRI fb SWI). Wheat yield in SRI fb SWI treatment was either similar (2014-15) or higher (2013-14 and 2015-16) than PTR fb CTW but was either similar (2013-14 and 2015-16) or lower (2014-15) than PTR fb ZTW. When comparing wheat grown after PTR, yields were higher (301 – 795 kg ha^−1^) under ZT than under CT. Similarly, when ZT wheat was grown after non-puddled rice (DSR or MTR), yields were similar in two out of three years (2013-14 and 2015-16) but in 2014-15, yield was 6% higher when grown after DSR than after MTR. Consistently across all years, wheat performed best when grown under ZT after DSR (DSR fb ZTW), followed by ZT wheat after MTR.

Wheat yields were 8% and 2% higher when grown after the shorter-duration rice cultivars PRH-10 (in year 1) and Arize-6129 (in year 3), respectively, relative to grown after the medium-duration rice cultivar Arize-6444. The rice cultivar did not affect wheat yield in year 2 (Table 4).

#### 3.1.3 Systems (Rice + wheat)

The system level yields were similar under different TCE methods in 2013-14 and 2014-15, but in 2015-16, the system level yields decreased in the following order: MTR fb ZTW (12.2 t ha^−1^) > DSR fb ZTW = SRI fb SWI (11.0 t ha) > PTR fb CTW (10.6 t ha^−1^). The system level yield of PTR fb ZTW was not different from those of SRI fb SWI and PTR fb CTW (Table 5).

**Table 5:** System yields (rice + wheat) under different tillage and crop establishment (CE) methods from 2013-14 to 2015-16 at PUSA in Samastipur, Bihar^1^.

^1^Within column for each year, means followed by the same letter are not different at the 0.05 level of probability using Tukey's HSD test.

*PTR fb CTW = Puddled transplanted rice followed by conventional tillage wheat; MTR fb ZTW = machine transplanted rice followed by zero-tillage wheat; DSR fb ZTW = dry seeded rice followed by zero-tillage wheat; SRI fb SWI = system of rice intensification followed by system of wheat intensification.

** Rice cultivar PRH-10 was used in 2013 and 2014, whereas in 2015, Arize-6129 was used as a short duration hybrid instead of PRH-10.

Across all TCE methods, the system level yield was 8.5 %, 12.6 %, and 2.9 % higher when the rice hybrid Arize-6444 was used relative to the rice hybrid PRH-10 in years 2013-14 and 2014-15 and Arize-6129 in the year 2015-16 (Table 5).

### 3.2 Economic analysis

#### 3.2.1 Cost of production and key input use

##### 3.2.1.1 Rice

**Fig. 2:**
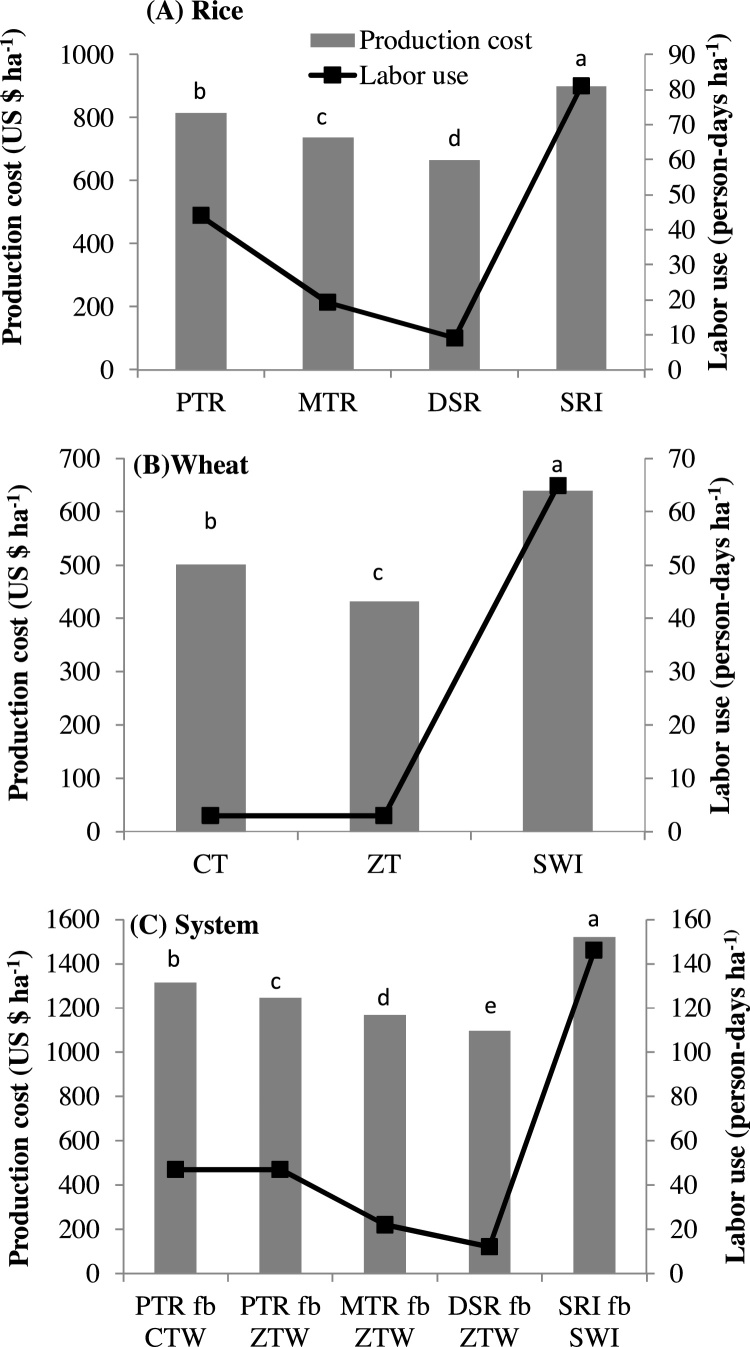
Total cost of production and labor use in different tillage and crop establishment methods of rice (A), wheat (B) and at the system level (C). Different letters indicate significant differences among treatments for production cost at 0.05 level of probability using Tukey’s HSD test of mean comparison. PTR = Puddled transplanted rice; MTR = machine transplanted rice; DSR = dry seeded rice; SRI = system of rice intensification; CT = conventional tillage; ZT = zero-tillage; SWI = system of wheat intensification

In rice, the total cost of production varied in the following order: SRI > PTR > MTR > DSR (Fig. 2a). Compared to conventional practice (PTR), the total cost of production in MTR and DSR was lower by US $ 77 and 149 ha^−1^, respectively. In contrast, the total cost of production increased by US$ 84 ha^−1^ in SRI relative to PTR.

The land preparation cost was US$ 72–91 ha^−1^ higher in puddled rice (PTR and SWI) than non-puddled rice (MTR and DSR) (Table 6). However, seed cost in non-puddled rice (DSR and MTR) was US $ 29 ha^-1^ higher than PTR and US$ 58 ha^−1^ than SRI. This was mainly because of the lower seed rate in PTR (12.5 kg ha^-1^) and SRI (5 kg ha^-1^) relative to DSR and MTR (20 kg ha^-1^ in both methods). There was no nursery cost involved in DSR, but among transplanted rice treatments, the nursery raising cost was highest in MTR (US$ 52 ha^-1^) followed by SRI (US$ 42 ha^-1^) and lowest in PTR (US$ 28 ha^-1^). The higher nursery raising cost in MTR was mainly due to (1) the higher labor use in the mat-type nursery for bed preparation and sieving of soil, (2) the additional cost of compost (1.25 t ha^-1^), and (3) slightly higher irrigation cost. Similarly, in SRI, the higher nursery cost relative to PTR was mainly due to slightly higher labor use in seedbed preparation and extra cost for compost (1.25 t ha^-1^). The crop establishment cost was highest in SRI followed by PTR and lowest in DSR. Compared to PTR, the cost of rice establishment was US $ 50 and US$ 64 ha^−1^ lower for the MTR and DSR methods, respectively, whereas the cost was US$ 67 ha^-1^ higher for SRI than PTR. The cost of nutrient management was US $ 21 ha^−1^ higher in SRI because of the additional vermicompost cost. The weed control cost was highest for DSR followed by SRI and was lowest in PTR and MTR. Compared to conventional practice (PTR), overall labor use was 37 person-days ha^-1^ higher in SRI but was 35 person-days ha^−1^ lower in DSR and 25 person-days ha^-1^ lower in MTR.

**Table 6:** Comparison of input cost and input use for key parameters under different rice tillage and crop establishment methods^1^.

Input parameter	PTR	MTR	DSR	SRI
Land preparation cost (USD ha^−1^)^a^	133	61	42	133
Seed cost (USD ha^−1^)	48	77	77	19
Nursery raising (USD ha^−1^)^b^	28	52	0	42
Crop establishment cost (USD ha^−1^)^c^	101	51	37	168
Compost and fertilizer cost (USD ha^−1^)	107	107	107	128
Weed management cost (USD ha^−1^)^d^	55	55	65	61
Seed rate (kg ha^−1^)	12.5	20	20	5
Labor input (person-days ha^−1^)^e^	44	19	9	81

^a^ Includes cost of dry tillage, wet tillage (puddling) and irrigation cost associated with wet tillage.

^b^ Includes all costs except seed cost. In SRI and MTR, the cost of vermicompost used in the nursery is also included.

c Includes seedling uprooting, transplanting, and rental charges for machines.

^d^ Includes herbicide cost, application cost and labor cost in weeding.

e Includes labor for nursery raising, seedling uprooting, transplanting/seeding and weed management only.

^1^ 1 USD = 65 INR.

Note: PTR = Puddled transplanted rice; MTR = machine transplanted rice; DSR = dry seeded rice; SRI = system of rice intensification.

##### 3.2.1.2 Wheat

The total cost of production for ZT wheat was US$ 69 ha^−1^ lower, whereas for SWI it was US$ 139 ha^-1^ higher than that for CT (Fig. 2b).

The land preparation cost for CT and SWI was US$ 86 ha^−1^ higher than ZT, whereas seed cost in SWI was US$ 46 ha^−1^ lower than CT and ZT wheat (Table 7). In terms of key inputs, total labor use was 62 person-days ha^−1^ higher in SWI, but the seed rate was lower by 75 kg ha^-1^ than the CT and ZT methods.

**Table 7:** Comparison of input cost and input use for key parameters under different wheat tillage and crop establishment (TCE) methods^1^.

Input parameter	CT	ZT	SWI
Land preparation cost (USD ha^−1^)^a^	85	0	85
Seed cost ((USD ha^−1^)	62	62	15
Seeding cost (USD ha^−1^)^b^	34	34	76
Compost and fertilizer cost (USD ha^−1^)	107	107	128
Weed management cost ((USD ha^−1^)^c^	31	31	122
Seed rate (kg ha^−1^)	100	100	25
Labor use in seeding and weed control (person-days ha^−1^)	3	3	65

^a^ Includes tillage and burndown herbicide (e.g., glyphosate) in ZT plots.

^b^ Includes rental charges of seed drill for ZT and CT plots and labor involved in SWI.

^c^ Includes herbicide cost, application cost and labor cost for weeding only.

^1^ 1 USD = 65 INR.

Note: CT = conventional tillage; ZT = zero-tillage; SWI = system of wheat intensification.

##### 3.2.1.3 System

At the system level, the total cost of production in different TCE methods decreased in the following order: SRI fb SWI > PTR fb CTW > PTR fb ZTW > MTR fb ZTW > DSR fb ZTW (Fig. 2c). In comparison to the conventional system (PTR fb CTW), total costs of production were reduced by US$ 146 ha^−1^ in MTR fb ZTW and US$ 217 ha^−1^ in DSR fb ZTW. SRI fb SWI resulted in a cost increase of US$ 204 ha^−1^ relative to PTR fb CTW.

#### 3.2.2 Gross margin (GM) and B: C ratio

##### 3.2.2.1 Gross margin

###### 3.2.2.1.1 Rice

TCE methods significantly influenced the gross margin in rice, wheat, and at the system level, whereas the effect of rice cultivars was variable with crops and years (Table 8).

**Table 8:** Net income and cost: benefit ratio from rice, wheat and system under different tillage and crop establishment (CE) methods from 2013-14 to 2016-17 at PUSA Samastipur in Bihar, India^1^.

^1^Within columns for each year, each crop, and TCE and cultivars within each year and each crop means followed by the same letter are not different at the 0.05 level of probability using Tukey's HSD test. Multiple mean comparisons were performed if ANOVA was significant.

*The exchange rate used, 1 USD = 65 INR.

Note: PTR fb CTW = Puddled transplanted rice followed by conventional tillage wheat; MTR fb ZTW = machine transplanted rice followed by zero-tillage wheat; DSR fb ZTW = dry seeded rice followed by zero-tillage wheat; SRI fb SWI = system of rice intensification followed by system of wheat intensification.

For rice, MTR resulted in the maximum gross margin and SRI the lowest. MTR gave a US$ 185 to 232 ha^−1^ higher gross margin relative to SRI in all the three study years. DSR and PTR did not differ from MTR in gross margin in the first two years (2013 and 2014) but, in the third year (2015), returned a US$ 176−181 ha^−1^ lower gross margin. Rice cultivars did not affect gross margin in the first two years when Arize-6444 (medium-duration hybrid) and PRH-10 (shorter-duration hybrid) were used, but in year 3, when Arize 6129 (shorter-duration hybrid) was used instead of PRH-10, rice cultivar significantly lowered the gross margin by US $ 58 ha^-1^.

###### 3.2.2.1.2 Wheat

In all the years, ZT wheat after non-puddled rice resulted in a higher gross margin relative to CT wheat or SWI (Table 8). In all years, SWI gave the lowest gross margin among all of the studied TCE methods. Compared to conventional tillage wheat (PTR fb CTW), ZT wheat after non-puddled rice (MTR fb ZTW and DSR fb ZTW) had US$ 196–270, US$ 138–177, and US$ 340−355 ha^−1^ higher gross margins in years 1, 2 and 3, respectively. In contrast, SWI (SRI fb SWI) resulted in US$ 139 and a 155 ha^-1^ lower gross margin in years 2 and 3 relative to conventional practice (PTR fb CTW). ZT wheat after PTR in all years had a higher gross margin (US$ 83−239 ha^-1^ depending on the year) than CT wheat.

###### 3.2.2.1.3 System (Rice + Wheat)

At the system level, alternate TCE methods such as MTR fb ZTW, DSR fb ZTW, and PTR fb ZTW gave a higher gross margin, but SRI fb SWI resulted in a lower gross margin than conventional methods (PTR fb CTW) (Table 8). In all years, MTR fb ZTW had the highest gross margin, and SRI fb SWI had lowest. DSR fb ZTW did not differ from MTR fb ZTW in gross margin in the first two years, but in the third year (2015-16), DSR fb ZTW had a US$ 167 ha^−1^ lower gross margin than MTR fb ZTW. PTR fb ZTW gave a higher gross margin than PTR fb CTW. PTR fb CTW and SRI fb SWI did not differ in gross margin in year 1, but in other years, the gross margin of SRI fb SWI was US$ 104 to 185 ha^-1^ lower than PTR fb CTW. Across the years, the gross margin in non-puddled rice followed by ZTW (MTR fb ZTW and DSR fb ZTW) was US$ 300−613 ha^-1^ and US$ 210−509 ha^-1^ higher than SRI fb SWI and PTR-CTW, respectively.

##### 3.2.2.2 B: C ratio

In rice, the B: C ratio was significantly influenced by TCE methods but not by rice cultivar (Table 8). The effect of TCE methods was variable over the years, but in general, the B: C ratio was highest in MTR and lowest in SRI. In year 1, DSR and MTR had higher B: C ratios than SRI but did not differ from PTR. In year 2, MTR had a higher B: C ratio than PTR and SRI but did not differ from DSR. In year 3, the B: C ratio decreased in the following order: MTR > DSR > PTR > SRI.

In wheat, in all years, the B: C ratio was higher in ZT than in CT and SWI (Table 8). Within ZT wheat, the B: C ratio did not differ when grown after DSR or MTR in year 1 and year 3 but was higher in ZT wheat after DSR than in ZT wheat after MTR in year 2. The B: C ratio in ZT wheat after PTR was always higher than that in CT wheat after PTR and SWI after SRI but lower than ZT wheat after DSR or MTR in years 2 and 3, although similar in year 1. Wheat had a higher B: C ratio when grown after lower-yielding shorter duration rice hybrids than when grown after high-yielding medium-duration hybrids in years 1 and 3. No effect of rice cultivars was observed on the B: C ratio in year 2.

At the system level, the B: C ratio decreased in the following order: MTR fb ZTW = DSR fb ZTW > PTR fb CTW > SRI fb SWI (Table 8). The rice variety had no effect on the B: C ratio at the system level in two out of three years (2014-15 and 2015-16), but in the first year (2013-14), the B: C ratio was higher with a shorter duration rice hybrid than with a medium-duration rice hybrid.

## 4 Discussion

### 4.1 Crop and cropping system yield

Rice yields did not differ with rice establishment methods in the first two years (2013 and 2014), but in the third year (2015), the yield of MTR was higher, SRI was similar, and DSR was lower than the conventional practice of PTR (Table 3). These results suggest that when different rice TCE methods are managed with similar levels of input, all methods have the potential to produce similar grain yields. In contrast to the claims of several researchers (Fernandes and Uhoff, 2002; Raj et al., 2017; Satyanarayana et al., 2007; Stoop et al., 2002), we did not find any yield advantage for SRI relative to other intensively managed rice production systems. It is important to note that most reports of higher yields with SRI contrast performance to farmers’ managed PTR without controlling for differences in plot-level fertility or input use (Berkhout et al., 2015). Studies that compared SRI with best management practices (BMPs) have observed variable results with some studies that did not find any yield advantage with SRI, as was the case in this study (Choudhury et al., 2007; McDonald et al., 2006), while others found lower (Sarala and Chellappan, 2011; Mazid et al., 2003) or higher yields in SRI than BMPs of PTR (Dass et al., 2016; Raj et al., 2017; Thakur et al., 2014).

A possible reason for the higher yield in MTR than PTR in the third year could be due to a better plant density (25 hills m^−2^) for high yield, as observed by Thakur et al. (2010), and a uniform shallow depth (∼ 2-cm) facilitated by a mechanical transplanter. Our results are consistent with many other researchers who also observed similar or higher (Farooq et al., 2001; Hossen et al., 2018; Kamboj et al., 2013) yield in MTR relative to PTR or SRI. Our results showing lower DSR yield in third year are similar to those of Kumar et al. (2018), who also reported lower yield in rice yields in DSR relative to PTR in the fourth and fifth years in rice-wheat systems in India. The yield decline in aerobic DSR relative to flooded transplanted rice was also observed with time in a long-term experiment conducted at the International Rice Research Institute (Peng et al., 2006). Researchers have reported soil sickness, higher weed competition, biotic stresses such as nematodes and rice mealybug, potential of mild water stress because of non-puddled condition leading to higher percolation rates, higher spikelet sterility, and lack of suitable cultivars are possible factors responsible for lower yield in DSR (Gathala et al., 2011b; Kumar and Ladha, 2011; Kumar et al., 2018; Kreye et al., 2009; Ladha et al., 2009; Mishra et al., 2019). Higher weed competition was not a factor in this study as plots were kept free from weed competition. One of the possible causes of a lower yield in this study was higher spikelet sterility (10 % in PTR versus 15 % in DSR; data not shown). The higher spikelet sterility in third year could be attributed to mild moisture stress and higher temperature stress during grain development phase. In the third year, rice was planted relatively late (21 June instead of 10 or 14 June) which pushed grain development into October when rainfall was negligible and maximum temperature (33 °C) was 1.6–2.8 °C higher than other two years of study which might have resulted in higher spikelet sterility in DSR. Despite supplemental irrigation, there can be mild moisture stress in DSR if the soil is lighter in nature (as the case in the study). The other possible causes could include biotic stress such as nematodes, which are associated with drier conditions and lighter soil (sandy loam) but were not verified in this study. These findings suggest the need for additional research to identify solutions for the long-term stability of DSR yields. Rice varieties with deep root systems, the use of nitrogen fertilizer as ammonium sulfate to buffer the effect of increased pH associated with continuous aerobic DSR, and rotating DSR with PTR have been suggested to reverse yield declines (Nie et al., 2009a, 2009b, Nie et al., 2012).

In this study, rice yields were highest in 2013 followed by 2015 and were lowest in 2014, irrespective of experimental treatment (Table 3). The higher yields in years 1 and 3 could be partly due to higher solar radiation in the months of July, August and September in year 1 than in year 2 (1484 MJ in 2013 versus 1295 MJ in 2014) and throughout the rice-growing season (June to October) in year 3 than in year 2 (Fig. 1c). Additionally, the high rice yields in year 1 could be explained because the experimental site was fallow for a season prior to the first rice season, potentially increasing indigenous soil fertility.

This study clearly demonstrated the positive effect on wheat yield with zero tillage establishment and non-puddled rice (Table 4). Based on a three-year average, ZTW after non-puddled rice (DSR fb ZTW or MTR fb ZTW) gave 660 kg–896 kg ha^−1^ higher yield than CTW. Wheat yield after PTR under ZT was on average 416 kg ha^−1^ (ranged from 301 to 795 kg ha^−1^) higher than that under CT. Zero tillage for wheat and the absence of soil puddling during the rice phase have been reported to have a positive impact on wheat yield because of improvement in soil physical properties and reduced impact of terminal heat stress during the grain filling stage (Gathala et al., 2011a, b; Kumari et al., 2011). ZT mitigates the effect of terminal heat stress by keeping the canopy cooler due to improved soil moisture retention (Gathala et al., 2011a). Based on a random survey of 1000 farmers from 40 villages in Bihar, India, an additional yield gain of 498 kg ha^−1^ (19 %) was recorded with the adoption of ZTW relative to CTW (Keil et al., 2015). In addition, this technology facilitates early wheat sowing by eliminating the time required for field preparation; early wheat sowing reduces the risk of terminal heat stress (CSISA, 2016; Kumar et al., 2018). The cause of lower yield in wheat after puddled rice relative to that after non-puddled rice has been has been attributed to poor rooting due to compaction (Gathala et al., 2011b; Kumari et al., 2011). Similarly, Kumar and Ladha (2011) summarized the impact of puddling in the rice phase on wheat yield and observed a 9% reduction in wheat yield when grown after puddled rice relative to grown after non-puddled rice.

We also observed that the yield advantage for ZTW after non-puddled rice was much higher than CTW (PTR fb CTW) in 2015-15 and 2015-16 when the months of February and March (during grain filling) were warmer than in 2013-14 (Table 4 and Fig. 1). The monthly average maximum temperature was 4.3 °C and 2.6 °C higher in February and March, respectively, in year 3 than year 1. In year 2, the monthly average maximum temperature was 2.3 °C higher in February than in year 1. The average minimum temperature was also higher in years 2 and 3 than year 1 in both February and March. These results suggest that ZTW, when grown after non-puddled rice, increases resilience to a warmer spring, which is anticipated to become more common with progressive climate change.

The SWI method did show some yield advantage over CTW in two out of three years. This could be partly due to the vermicompost used in SWI, which can improve soil physical properties and nutrients stocks. However, SWI did not show any yield advantage over ZTW when grown after PTR and performed poorly relative to ZTW when grown after non-puddled rice. In this study, we also observed that the yield level in the rice phase can influence the yield of the succeeding wheat crop. Wheat yields were lower when grown after high yielding medium duration rice than when grown after a lower yielding shorter duration rice. The higher wheat yields observed following shorter duration rice could be related to differences in soil fertility, with the medium duration and higher yielding hybrid extracting more nutrients from the system. These results suggest the need for more research on optimal nutrient management by considering both crops in the annual rotation.

At the system level, all TCE methods had similar yields except in the third year, when yields were higher in MTR fb ZTW (Table 5). Despite the lower yield in DSR in year 3 than the rest of the rice establishment methods, the system level yield of DSR fb ZT was higher than that of conventional practice (PTR fb ZTW) or similar to SRI fb SWI, mainly because it compensated the loss in rice with a gain in wheat yield. Additionally, rice cultivars influenced the system yield. Despite lower wheat yield after the high yielding rice hybrid Arize-6444 (0.1 to 0.4 t ha^−1^), the system level yields were higher with the medium-duration rice hybrid (Arize-644) than the short-duration rice hybrid (PRH-10/Arize-6129) because the gain in wheat yield was not fully offset by lower rice yields (0.4 to 1.4 t ha^−1^) in rice.

### 4.2 Economic analysis

In rice, despite similar yields with different rice establishment methods, the higher gross margin and B: C ratio in MTR relative to SRI and PTR were due to lower production costs (Table 8; Fig. 2). The lower production costs also resulted in a higher B:C ratio in DSR. The lower production costs in MTR and DSR were mainly attributable to savings in land preparation and crop establishment costs, as wet tillage (puddling) and manual transplanting were omitted in both DSR and MTR. The savings in land preparation and crop establishment costs in DSR were US$ 155 and US$ 222 ha^−1^ relative to PTR and SRI, respectively, whereas these savings in MTR were US$ 122 and US$ 189 ha^−1^. In contrast, SRI gave the lowest gross margin and B:C ratio because of higher production costs associated with higher labor use in transplanting and cono-weeding operations and extra costs for nursery raising and fertilizer management due to the inclusion of vermicompost, despite a drastic reduction in seed cost because of the low seed rate (5 kg ha^−1^ versus 20 kg ha^−1^ in MTR and DSR and 12.5 kg ha^−1^ in PTR). These results demonstrate that MTR and DSR can provide similar or higher yields and profitability but with lower investments in the cost of cultivation. Moser and Barrett (2003) and Takahashi and Barrett (2013) also reported an increase in labor use with the SRI method. Serpantié and Rakotondramanana (2013) argued that the small increase in rice yield in SRI is negated by the overall increase in labor use resulting in no change in labor productivity. Rakotomalala (1997) observed 62 % extra labor in weeding and 17 % extra labor in transplanting operations in the SRI method. We also observed higher labor use in transplanting (28 versus 45 person-days ha^−1^) and weeding (4 versus 20 person-days ha^−1^) operations in SRI relative to PTR. Overall, 37, 62, and 72 person-days ha^−1^ extra labor were used in SRI relative to PTR, MTR, and DSR, respectively. Our study finds that SRI is more labor- and capital-intensive than other TCE methods.

Although the yield of PRH-10 was lower (∼1.3 t ha^−1^) than that of Arize-6444, these cultivars did not differ in gross margin and B:C ratio. This was because of a combination of (1) the higher market price of PRH-10 than Arize-6444, as PRH-10 is a superfine aromatic rice hybrid of basmati quality (Das et al., 2017) that fetches a premium price, and (2) the lower cost of cultivation, especially due to the cheaper seed cost of PRH-10 (INR 120 versus INR 250 kg^−1^) and savings of one irrigation cost because its duration is shorter than that of Arize-6444. The market price of PRH-10 was INR 2000 (US$ 31) per ton higher than that of Arize-6444. However, Arize-6129 and Arize-6444 did not differ in their market price and seed cost, and the lower yield of Arize-6129 was also reflected in the lower gross margin and B:C ratio relative to Arize-6444 in the third year.

In wheat, SWI was the most capital-intensive method, followed by CTW and ZTW. The cost of cultivation of SWI was 28 % higher than CTW and 48 % higher than ZTW (Table 7). The higher cost of cultivation of SWI relative to CTW and ZTW was mainly because of the extra labor cost involved in seeding by manual dibbling and labor use for the cono-weeder and the extra cost for the vermicompost. These extra costs exceed the savings in seed cost due to the low seed rate in SWI. Additionally, in ZT, there was savings of land preparation costs due to the avoidance of tillage for land preparation. The higher gross margin and B:C ratio in the ZTW methods (PTR fb ZTW or DSR fb ZTW or MTR fb ZTW) than the CTW and SRI methods were due to either the lower cost or combination of lower cost and higher yield (Table 8). ZTW resulted in an additional gross margin of US$ 83−239 ha^−1^ and US$ 138−355 ha^−1^ when grown after puddled and non-puddled rice, respectively, in comparison to CTW (Table 8). From their survey of 1000 farmers, Keil et al. (2015) recorded a total economic gain of US$ 110 ha^-1^ from ZTW relative to CTW as a result of yield increase and cost reduction. Because of the clear advantages of ZTW in terms of productivity, profitability, and resilience to heat stress (CSISA, 2015; Erenstein and Laxmi, 2008; Keil et al., 2015), it has been widely adopted in northwest India and recently in the eastern IGP, especially in Bihar and eastern UP (CSISA, 2010, 2017). Among different TCE methods, SWI was the least profitable and most capital-intensive.

**Fig. 3:**
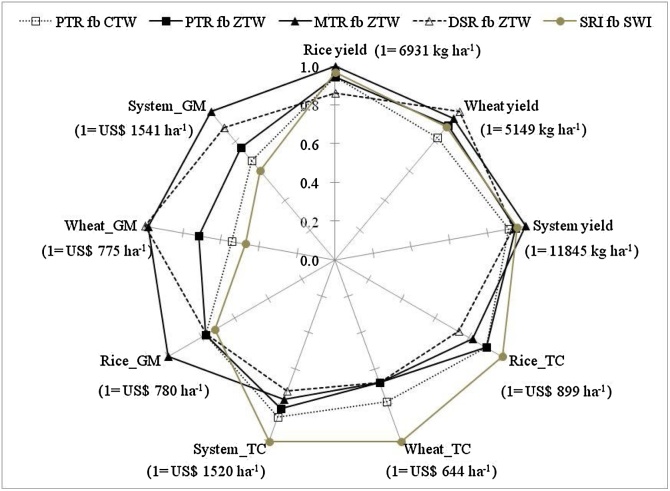
Productivity and economic indicators of different tillage and crop establishment methods based on a three year average (2013-14 to 2015-16). Variables included are yield of rice, wheat, and system; total cost (TC) of production in rice, wheat, and at the system level; and gross margin (GM) in rice, wheat and at the system level. Variable means are normalized on a 0–1 scale, with 1 representing the highest absolute value of that variable. The highest absolute value is also shown for each parameter.

In summary, the results of this three-year study clearly demonstrated the benefits and trade-offs associated with alternate TCE methods in terms of the crop and cropping system yields and profitability. The impact of these alternate TCE methods on grain yield was greater for wheat than rice but did significantly influence the cost of production and gross margin at the crop and cropping systems levels (Fig. 3). Based on a three-year average, although rice yield was lower in DSR fb ZTW than the other TCE methods, it was compensated by wheat with the highest yield, which resulted in system level grain yields similar to other TCE methods. MTR fb ZTW and DSR fb ZTW were more profitable than the conventional practice of PTR fb CTW because of the lower cost of production and higher wheat yields. In contrast, SRI fb SWI, despite similar system yields relative to other TCE methods, was the least profitable because of the high cost of production associated with SRI and SWI. These results suggest that MTR fb ZTW and DSR fb ZTW have the potential to improve the productivity and profitability of farmers in rice-wheat systems in the eastern IGP. Despite savings in production costs and higher profitability with potential to give similar or higher yields, adoption of DSR and MTR is low (CSISA, 2016). This could be due to the lack of availability of appropriate machines (transplanters and seed drills), one of the major constraints in the adoption of capital-intensive technologies among smallholders (CSISA, 2016). Efforts are needed to strengthen the service economy for scale-appropriate mechanization to increase the access of smallholders to these capital-intensive technologies. For DSR, other adoption constraints include risks of (1) poor and uneven crop establishment due to stand mortality attributed to inundation caused by untimely rain during crop emergence, (2) high weed incidence, and (3) long-term yield decline (Kumar and Ladha, 2011; Kumar et al., 2018). More research is needed in these areas to overcome these constraints to take full advantage of the cost-effective and resource-efficient DSR method.

## 5 Limitation of the study

To enhance our results, future studies should endeavor to more carefully measure the amount of irrigation water applied. Moreover, this study also did not assess the performance of these alternatives in terms of energy input, greenhouse gas (GHG) emissions, and changes in soil parameters (soil health). To fully ascertain impacts on sustainability, additional estimates of energy use, GHG emissions, and soil health are required.

## 6 Conclusions

Our results demonstrated that alternative tillage and crop establishment methods such as MTR and DSR in non-puddled conditions during rice and zero-tillage in wheat can reduce the cost of production with a similar or higher yield, which ultimately leads to higher farm profitability of rice-wheat systems in the EIGP compared to the current practice of puddled transplanted rice and conventional tillage wheat. The combination of avoiding puddling during the rice phase and ZT in wheat had a positive impact on wheat yield, which was higher in years with a warmer spring. The results indicate that these alternative methods have the potential to enhance tolerance to terminal heat stress. However, more research is needed to understand the system changes with continuous DSR to make DSR stable over time. In addition, to scale out these profitable alternative methods, there is a need to strengthen the service economy around mechanized planting, which will enhance access to these capital-intensive technologies for smallholders through service provision.

## Declaration of interests

The authors declare that they have no known competing financial interests or personal relationships that could have appeared to influence the work reported in this paper.

## CRediT authorship contribution statement

**Madhulika Singh:** Investigation, Data curation, Writing - original draft. **Pankaj Kumar:** Investigation, Data curation, Writing - review & editing. **Virender Kumar:** Conceptualization, Formal analysis, Funding acquisition, Writing - original draft, Supervision, Visualization. **I.S. Solanki:** Conceptualization, Supervision. **Andrew J. McDonald:** Conceptualization, Funding acquisition, Writing - review & editing. **Ajay Kumar:** Investigation, Writing - review & editing. **S.P. Poonia:** Data curation, Writing - review & editing. **Vipin Kumar:** Writing - review & editing. **Anurag Ajay:** Writing - review & editing. **Anurag Kumar:** Writing - review & editing. **Deepak K. Singh:** Writing - review & editing.Writing - review & editing, Funding acquisition. **Sudhanshu Singh:** Writing - review & editing, Project administration. **Ram K. Malik:** Conceptualization, Funding acquisition, Writing - original draft, Supervision.
